# Exercise for patients with major depression: a protocol for a systematic review with meta-analysis and trial sequential analysis

**DOI:** 10.1186/s13643-015-0030-6

**Published:** 2015-04-02

**Authors:** Jesper Krogh, Helene Speyer, Christian Gluud, Merete Nordentoft

**Affiliations:** Mental Health Centre Copenhagen, Faculty of Health Sciences, University of Copenhagen, Bispebjerg Bakke 23, opg. 13a, DK-2400 Copenhagen, Denmark; Copenhagen Trial Unit, Centre for Clinical Intervention Research, Rigshospitalet, Copenhagen University Hospital, Blegdamsvej 9, DK-2100 Copenhagen Ø, Denmark

**Keywords:** Major depression, Depression, Exercise, Physical activity, Systematic review, Aerobic exercise, Strength training

## Abstract

**Background:**

The lifetime prevalence of major depression is estimated to affect 17% of the population and is considered the second largest health-care problem globally in terms of the number of years lived with disability. The effects of most antidepressant treatments are poor; therefore, exercise has been assessed in a number of randomized clinical trials. A number of reviews have previously analyzed these trials; however, none of these reviews have addresses the effect of exercise for adults diagnosed with major depression.

**Methods/design:**

The objective of this systematic review is to investigate the beneficial and harmful effects of exercise, in terms of severity of depression, lack of remission, suicide, and so on, compared with treatment as usual with or without co-interventions in randomized clinical trials involving adults with a clinical diagnosis of major depression. A meta-analysis of the effect estimates of the individual trials, taking bias risk into consideration, will be carried out. Any heterogeneity will be explored using meta-regression and subgroup analyses. Trial sequential analysis will be carried out on the trials to control for risks of random errors. The results from the study will aid health authorities and clinicians to understand whether exercise should be offered to patients with major depression.

## Background

Depression is a common disease affecting up to 17% of the population during their lifetime [1]. Based on data from the WHO, depression is thought to be the second largest health-care problem globally, in terms of years lived with disability (YLD) [2]. Depression is also observed as a co-morbidity in a number of somatic diseases, significantly contributing poorer outcomes in diseases such as cancer, ischaemic heart disease, and diabetes. Depending on its severity, depression is often treated using psychotherapy, antidepressants, or a combination of both. However, the clinical efficacy of antidepressants [3,4] and psychotherapy [5-7] has been challenged. Both treatments are costly in terms of time and money and may also have adverse effects. Compliance with antidepressant treatment is poor; the dropout rate in clinical trials is reported to be between 12% and 40% within the initial 6 to 8 weeks of treatment [3,8].

The weakness of evidence for the beneficial effect of treatment, along with problems related to cost, harm, and low compliance, has resulted in an interest in using alternative or complementary therapies. The use of exercise as an intervention has attracted a lot of attention, and various forms of exercise varying in intensity have been assessed in a number of randomized clinical trials to test their effectiveness as a treatment for patients with depression.

In 2011, the authors of this paper published a meta-analysis of randomized clinical trials examining the effect of exercise on depressive symptoms in patients with clinical depression [9]. The results suggested that referring patients with clinical depression to exercise programs was associated with a small to moderate effect on depressive symptoms. However, restricting the analysis to three trials with a low risk of bias, the effect estimate was non-significant. Since 2011, other reviews have been published on the effect of exercise on depressive symptoms [10], in older people [11] and in patients with chronic illnesses [12]. However, none of these reviews addressed the specific population of adults diagnosed with major depression according to valid diagnostic criteria, such as the International Classification of Diseases [13] or the Diagnostic and Statistical Manual of Mental Disorders [14]. The reviews contained a number of trials that included volunteers who were defined as being depressed on the basis of psychometric testing (for example, Beck Depression Inventory [15]), as opposed to individuals with a clinical diagnosis of major depression. Furthermore, several randomized clinical trials investigating the effect of exercise in clinically depressed individuals have been published since our 2011 review.

### Objectives

The objective of this present systematic review is to investigate the beneficial and harmful effects of exercise, in terms of severity of depression, lack of remission, quality of life, suicide, and so on, compared with or without co-interventions in adults with a clinical diagnosis of major depression.

Apart from including new trials, the current systematic review differs from the previous study [9]. The current review only considers trials including participants with a diagnosis of major depression and does not include patients referred with depressive symptoms. The harmful effects of exercise interventions are also addressed, and bibliographical searches have been extended to include a Chinese and a South-American database.

## Methods/design

- This systematic review will only include randomized clinical trials. This protocol is not registered with PROSPERO.

### Inclusion criteria

- Participants should be diagnosed as having major depression according to a valid and recognized diagnostic system (that is, Research Diagnostic Criteria (RDC) [16], International Classification of Diseases (ICD) [13], or Diagnostic and Statistical Manual of Mental disorders (DSM) [14]).
- Participants aged >17 years of both sexes.
- Randomized clinical trials. A trial is defined as a randomized clinical trial if the allocation of participants to intervention and comparison groups is described as randomized (including terms such as ‘randomly’, ‘random’, and ‘randomization’).
- No restriction to type of publication (that is, we will include abstracts and full text reports).

### Exclusion criteria

- Trials measuring depression immediately after a single bout of exercise.
- Trials comparing one form of exercise *versus* another.
- Trials comparing different exercise intensities without including a control group.

### Interventions

- The trials had to allocate participants to an exercise intervention *versus* a control group (that is, exercise *versus* a control group receiving no intervention or treatment as usual or an attention control using light exercise) or using exercise as an add-on-treatment (that is, exercise plus medication in the experimental group *versus* medication alone in the control group).
- Exercise intervention is defined as a systematic physical intervention with the intention to increase muscle strength and/or cardiovascular fitness. A control group could include no treatment or only an attention control using light exercise. However, it should specifically be mentioned by the authors that the intervention is intended to be a control intervention. Light exercise would be equivalent to stretching or light aerobic exercise.

### Outcomes

The primary outcomes are 1) depressive symptoms measured on a continuous scale assessed at the end of the intervention; 2) lack of remission, that is, a binary outcome of the proportion of participants in each intervention group of the trial who did not obtain remission at the end of the intervention according to the authors’ own definition; and 3) serious adverse events defined according to ICH-GCP as any untoward medical occurrence that was life threatening, resulted in death or persistent or significant disability (ICH-GCP 1997). Serious adverse events will accordingly include suicide attempts as well as suicides. The secondary outcomes are non-serious adverse events, depressive symptoms, and lack of remission assessed beyond the intervention.

### Search strategy

The search will include search CENTRAL, MEDLINE, EMBASE, and Science Citation Index (Web of Science) using medical subject headings (MESH or similar) when possible and text word terms: depression, depressive disorder and exercise, aerobic, non-aerobic, physical activity, physical fitness, walking, jogging, running, bicycling, swimming, strength, and resistance. The search will also include LILAC (Latin American and Caribbean Health Sciences Literature) and the Chinese Wanfang database using text word terms: depression, depressive disorder, and exercise or physical training. The flow of trial reports and reasons for exclusion will be presented in the PRISMA flow chart and categorized: non-clinical populations (that is, not diagnosed according to a diagnostic system), review or commentary, not a randomized trial, acute exercise (that is, studies/trials investigating the effect of a single bout of exercise), and trials including patients with other psychiatric diagnoses (for example, bipolar). In addition, reference lists of relevant reviews will be searched for additional trials.

#### Study selection

One investigator (JK) will examine titles and abstracts to remove obviously irrelevant reports. Two investigators (JK + HS) will examine the remaining full text reports determining compliance with inclusion criteria.

### Data extraction

Two authors (JK, HS) will independently extract data using a pre-piloted structured form. Any discrepancies in the data extraction or inclusion/exclusion of trials will be resolved by referring to the original papers. CG or MN will assist as adjudicator in cases of disagreements. The authors will not be blinded to article results, authors, or institutions. Data extraction will, in addition to outcomes, include information regarding country of origin, number of randomized participants, number of participants included in efficacy analysis, mean age of participants, diagnostic system, baseline assessment of depression severity, type of intervention, frequency of intervention, , duration of intervention, and recruitment setting (clinical *vs*. non-clinical).

The authors JK, CG, and MN have previously published trial reports assessing the effect of exercise in patients with depression [17,18]. To avoid academic bias, a third assessor (CH) will assist HS in bias assessment for these two trials.

#### Risk of bias assessment

Methodological studies show that trials with unclear or inadequate methodological quality regarding bias domains may be associated with bias (systematic error, the overestimation of benefits, and the underestimation of harms) when compared to trials using adequate methodology [19-24]. Definitions in the assessment of bias risk of a trial will be done according to the Cochrane Handbook for Systematic Reviews of Interventions [19] of the following domains: allocation sequence generation, allocation concealment, blinding of participants and personnel, blinding of outcome assessors, incomplete outcome data, selective outcome reporting, for-profit bias, and other bias. Please see Appendix for specifications on bias assessment.

Trials assessed as having ‘low risk of bias’ in all of the above specified domains will be considered ‘trials with low risk of bias’. Trials assessed as having ‘uncertain risk of bias’ or ‘high risk of bias’ in one or more of the above specified domains shall be considered trials with ‘high risk of bias’. In line with our previous systematic review [9] and the latest Cochrane review [10], trials with low risk of bias in the allocation concealment domain, blinded outcome assessment domain, and the intention-to-treat analysis domain will also be characterized as trials with ‘lower risk of bias.’ However, in case no or few trials with low risk of bias will be included, we shall remember that the chance to know the ‘true’ intervention effect in trials with ‘lower risk of bias’ is low or absent.

### Data synthesis and analysis

In order to be able to include all of the studies in our meta-analysis [25], estimates of standardized mean difference (SMD) for each individual study will be carried out. SMD is the mean difference in depression score between the exercise and control groups dived by the pooled standard deviation. The result is a unit less effect size measure, which is comparable to other studies using other but similar measures of outcome. By convention, SMD effect sizes of 0.2, 0.4, and 0.8 are considered small, medium, and large, respectively. For dichotomous variables, we will calculate the relative risks with a 95% confidence interval. It is expected that some trials have several intervention groups. Data from the experimental groups will be pooled and compared with the data from the control group. In case of discrepancies between the random-effects model analysis and the fixed-effect model analysis, both results will be reported [26]; otherwise, only results from the random-effects analysis will be reported.

The degree of heterogeneity will be quantified using the *I*-squared statistic [27], which can be interpreted as the percentage of variation observed between the trials attributable to between-trial differences, rather than sampling error (chance). Heterogeneity will be explored by analysis of sub-groups (see below).

For the primary outcomes, trial sequential analysis will be attempted, based on mean differences or proportions [28,29]. In order to calculate the required information size and the cumulative Z-curve’s eventual breach of relevant trial sequential monitoring boundaries, the required information size for a primary continuous outcome will be based on type I error of 5%, a beta of 10%, the standard error of the meta-analysis, and a minimal difference of three points on the HAM-D_17_. In order to calculate the required information size and the cumulative Z-curve’s eventual breach of relevant trial sequential monitoring boundaries, the required information size for the primary dichotomous outcomes will be based on type I error of 5%, a beta of 10%, the proportion of patients in the control group with the outcome, and a relative risk reduction of 15% or 30%. Most systematic reviews do not contain sufficient power [30], and if there is no significant effect of the intervention, it is also interesting to know whether this represents an absence of evidence (the cumulative Z-curve has not reached the futility area), or if it represents evidence of an absence of effect (the cumulative Z-curve has reached the futility area). If an absence of evidence persists, the likely number of participants still needed to answer the question raised can also be assessed. An interesting question is whether the trial sequential monitoring boundaries for benefit (or potentially for harms) are crossed. This informs as to whether new trials should have been stopped. Bayes factors will be calculated for all primary values (the ratio between the *P* value probability divided by the probability of the meta-analysis result, given that an anticipated intervention effect is the true effect) [26].

To assess the potential impact of missing data (incomplete outcome data bias), a ‘best-worst’ case scenario will be assessed, assuming that all participants lost to follow-up in the intervention group had a beneficial outcome (the group mean minus 1 standard deviation (SD)), and all those with missing outcomes in the placebo group have had a harmful outcome (the group mean plus 1 SD and 2 SD). It is also planned to perform the reverse ‘worst-best-case’ scenario analysis [26].

Regarding the outcome of lack of remission, trials will be included with incomplete or missing data. In case of missing data for the ‘lack of remission’ outcome, missing values will be imputed in sensitivity analysis according to the following scenarios [31]: 1) poor outcome analysis: assuming that none of the drop-outs/participants lost from both the experimental and the control arms experienced the outcome, including all randomized participants in the denominator; 2) good outcome analysis: assuming that all of the drop-outs/participants lost from the experimental and the control arms experienced the outcome, including all randomized participants in the denominator; 3) extreme case analysis favoring the experimental intervention (‘best-worse’ case scenario): none of the drop-outs/participants lost from the experimental arm, but all of the drop-outs/participants lost from the control arm experienced the outcome, including all randomized participants in the denominator; and 4) extreme case analysis favoring the control (‘worst-best’ case scenario): all drop-outs/participants lost from the experimental arm, but none from the control arm experienced the outcome, including all randomized participants in the denominator.

#### Subgroup analyses

In subgroup analyses, the possible effects of a number of variables on outcomes and heterogeneity will be compared. It is expected that no, or very few, trials with low risk of bias will be found, and therefore, an assessment of the risk of bias by comparing trials with lower risk of bias is planned according to adequate allocation concealment, blinded outcome assessment, and intention-to-treat analysis to trials with high risk of bias according to these domains. The effect of age will be assessed by comparing trials including older participants (mean age >60 years) with trials including younger participants (mean age <60 years). The effect of group *versus* individual exercise will be assessed by comparing trials using group exercises compared to trials using individual exercises. The effect of the duration of intervention will be assessed by comparing trials with short duration of intervention to trials with long duration of intervention. The two groups formed will be based on the median duration of intervention employed. The effect of type of control group will be assessed by comparing trials with trials, using attention control to trials with other forms of control. Assessment of the effect of using exercise as an add-on therapy by comparing trials using placebo/attention control/TAU as control group to trials using antidepressant as a control group will be carried out. In addition, a within-study comparison of low-dose exercise *versus* high-dose exercise in trials using different exercise intensities will be performed. The effect of co-morbid somatic disease will be assessed by comparing the effect estimates from trials including patients with depression compared to trials including patients with depression in addition to a somatic disease.

Publication bias will be assessed by visual inspection of a funnel plot and by Egger’s test. The meta-analyzed results will be presented in a summary of findings table according to the GRADE system [32].

## Discussion

In this systematic review, the assessment of the benefits and harms of exercise interventions for adults with clinical diagnosis of major depression will be reviewed. It is intended to minimize selection bias by including bibliographical databases from South America (LILACS) and China (Wanfang) in addition to standard search strategies limited to western bibliographical databases (for example, CENTRAL, MEDLINE, EMBASE). In addition to meta-analysis, trial sequential analysis to assess our risks of random error is planned. The final discussion will include an analysis of the strength and limitations of the evidence and of the current review.

Based on the authors’ previous review and intimate knowledge of the current subject, we expect to include more than 1,000 patients diagnosed with depression included in randomized clinical trials. The current review will support health-care providers and decision makers within the health-care system on the decision to include exercise as a standard treatment for patients with depression.

## Appendix: Search strategy

We used MESH terms or similar and secondly text word terms.

1. Depression
2. Depressive disorder
3. Major Depression
4. Exercise
5. Aerobic
6. Non-aerobic
7. Physical activity
8. Physical fitness
9. Walk*
10. Jog*
11. Run*
12. Bicycling
13. Swim*
14. Strength
15. Resistance
16. 1 OR 2 OR 3
17. 4 OR 5 OR 6 OR 7 OR 8 OR 9 OR 10 OR 11 OR 12 OR 13 OR 14 OR 15
18. 16 AND 17

## Appendix: Bias assessment

### Allocation sequence generation

- Low risk of bias: sequence generation was achieved using computer random number generation or a random number table. Drawing lots, tossing a coin, shuffling cards, and throwing dice are adequate if performed by an independent person not otherwise involved in the trial.
- Uncertain risk of bias: the method of sequence generation was not specified.
- High risk of bias: the sequence generation method was not random.

### Allocation concealment

- Low risk of bias: the participant allocations could not have been foreseen in advance of, or during, enrolment. Allocation was controlled by a central and independent randomization unit. The allocation sequence was unknown to the investigators (for example, if the allocation sequence was hidden in sequentially numbered, opaque, and sealed envelopes).
- Uncertain risk of bias: the method used to conceal the allocation was not described so that intervention allocations may have been foreseen in advance of, or during, enrolment.
- High risk of bias: the allocation sequence was likely to be known to the investigators who assigned the participants.

## Blinding of participants and personnel

We do not expect this to happen in many trials, but in case investigators have tried to blind these groups, we will use the following:

- Low risk of bias: blinding was performed adequately.
- Uncertain risk of bias: there was insufficient information to assess whether blinding was likely to induce bias on the results.
- High risk of bias: no blinding or incomplete blinding.

## Blinding of outcome assessors

We do not expect this to happen in many trials, but in case investigators have tried to blind the outcome assessors, we will use the following:

- Low risk of bias: the assessment of outcomes was not likely to be influenced by lack of blinding.
- Uncertain risk of bias: there was insufficient information to assess whether blinding was likely to induce bias on the results.
- High risk of bias: the assessment of outcomes was likely to be influenced by lack of blinding.

### Incomplete outcome data

- Low risk of bias: missing data were unlikely to make treatment effects depart from plausible values. Sufficient methods, such as multiple imputation, have been employed to handle missing data.
- Uncertain risk of bias: there was insufficient information to assess whether missing data in combination with the method used to handle missing data were likely to induce bias on the results.
- High risk of bias: the results were likely to be biased due to missing data.

Analyses is considered intention to treat if missing data is handled by adequate methods (mixed models, multiple imputations, or similar) or if no missing data was encountered (last observation carried forward was considered inadequate).

### Selective outcome reporting

- Low risk: all predefined, or clinically relevant and reasonably expected, outcomes are reported on. If the original trial protocol is available, the outcomes should be those called for in that protocol. (Note: If the trial protocol is obtained from a trial registry (for example, www.clinicaltrials.gov), the outcomes to be sought are those enumerated in the original protocol if the trial protocol was registered before or at the time the trial has begun; if the trial protocol was registered after the trial has begun, those outcomes will not be considered to be reliable in representing the outcomes initially being sought.) If the trial protocol is not available (or if the protocol was registered after the trial was begun), the review authors will decide, when they are writing the protocol for the systematic review, what clinically relevant and reasonably expected outcomes would be and will explicitly state those outcomes in the pertinent methodology part of their protocol for the systematic review.
- Unclear risk: not all predefined, or clinically relevant and reasonably expected, outcomes are reported fully, or it is unclear whether data on these outcomes were recorded or not.
- High risk: one or more predefined or clinically relevant and reasonably expected outcomes were not reported, despite the fact that data on these outcomes should have been likely to have been available and even recorded.

### For-profit bias

- Low risk of bias: the trial appears to be free of industry sponsorship or other type of for-profit support that may manipulate the trial design, conductance, or results of the trial.
- Uncertain risk of bias: the trial may or may not be free of for-profit bias as no information on clinical trial support or sponsorship was provided.
- High risk of bias: the trial was sponsored by industry or received other type of for-profit support.

### Other bias

- Low risk of bias: the trial appears to be free of other components (for example, academic bias) that could put it at risk of bias.
- Uncertain risk of bias: the trial may or may not be free of other components that could put it at risk of bias.
- High risk of bias: there are other factors in the trial that could put it at risk of bias (for example, baseline imbalance or academic bias).

## Abbreviations

DSM: Diagnostic and Statistical Manual of Mental Disorders
HAM-D_17_: Hamilton Depression Rating Scale - 17 Items
ICD: International Classification of Diseases
ICH-GCP: International Conference on Harmonization of Technical Requirements for Registration of Pharmaceuticals for Human Use - Guideline for Good Clinical Practice
LILACS: Latin American and Caribbean Health Sciences Literature
RDC: Research Diagnostic Criteria
SMD: standardized mean difference
WHO: World Health Organization
YLD: years lived with disability
